# Ginsenoside Rh2 Enhances CD8^+^ T Cell-Mediated Anticancer Immunity in Hepatocellular Carcinoma

**DOI:** 10.3390/nu18142224

**Published:** 2026-07-08

**Authors:** Jinbum Park, Inae Jeong, Anna Han, Ok-Kyung Kim

**Affiliations:** 1 Division of Food and Nutrition, Chonnam National University, Gwangju 61186, Republic of Korea; pabre96@jnu.ac.kr; 2 Human Ecology Research Institute, Chonnam National University, Gwangju 61186, Republic of Korea; wjddlsdo2@jnu.ac.kr; 3 Department of Food Science and Human Nutrition, Jeonbuk National University, Jeonju 54896, Republic of Korea; annahan8659@jbnu.ac.kr; 4 K-Food Research Center, Jeonbuk National University, Jeonju 54896, Republic of Korea

**Keywords:** CD8^+^ T cell, ginsenoside Rh2, hepatocellular carcinoma, regulatory T cell, tumor microenvironment

## Abstract

**Background:** Ginsenoside Rh2 (Rh2), a bioactive metabolite of *Panax ginseng*, has documented anticancer effects, but its role in cancer–immune crosstalk remains incompletely defined. Thus, we aimed to investigate the role of Rh2 in hepatocellular carcinoma (HCC) cells and immune regulation. **Methods**: We used a co-culture system of murine Hepa1-6 hepatocellular carcinoma cells or nonmalignant AML12 hepatocytes with primary splenocytes to model cancer–immune interactions during Rh2 exposure. Readouts included cell viability, nuclear morphology, and multiparameter flow cytometry. **Results**: In a co-culture system of Hepa1-6 cells and splenocytes, Rh2 decreased bulk cell viability and increased apoptosis in Hepa1-6 cells. CD8^+^ T cells exhibited enhanced effector features, with increased CD107a and IFN-γ expression following Rh2 treatment. Rh2 reduced PD-L1 expression on Hepa1-6 cells and splenocytes, and PD-1 expression on CD8^+^ T cells. Rh2 also reduced TGF-β1 and IL-6 levels in both Hepa1-6 cells and splenocytes, and decreased IL-10 levels in splenocytes. This was accompanied by a reduction in CD4^+^CD25^+^FOXP3^+^ regulatory T cells (Tregs). **Conclusions**: In a physiologically relevant cancer–immune context, Rh2 reprograms suppressive interactions by enhancing CD8^+^ T cell effector function, dampening PD-L1/PD-1 signaling, and reducing key immunosuppressive cytokines and Tregs. These coordinated effects position Rh2 as a candidate multi-target immunomodulatory agent for enhancing anticancer immunity.

## 1. Introduction

Hepatocellular carcinoma (HCC) is a major global health burden and ranks among the top three causes of cancer-related deaths [1]. Metabolic dysfunction-associated steatotic liver disease (MASLD) has emerged as a leading etiology of HCC, paralleling the rising prevalence of obesity, type 2 diabetes, and dyslipidemia [2,3]. Despite recent advances in immunotherapy, treatment outcomes remain unsatisfactory for many patients because of the highly immunosuppressive tumor microenvironment (TME) that characterizes HCC [4,5]. The HCC microenvironment is enriched with immunosuppressive cytokines, including IL-6, IL-10, and TGF-β, and is frequently associated with the accumulation of regulatory T cells (Tregs), myeloid-derived suppressor cells (MDSCs), and other suppressive immune populations [6,7]. These factors impair cytotoxic CD8^+^ T-cell activity, promote immune evasion, and contribute to PD-1/programmed death-ligand 1 (PD-L1)-mediated T-cell exhaustion [8]. Consequently, considerable efforts have focused on developing strategies that restore antitumor immunity by reversing immunosuppressive signaling within the HCC microenvironment [9,10,11,12].

In this context, naturally derived agents with immunomodulatory potential—owing to low toxicity and multitarget activity—have garnered particular interest [13,14]. Among them, *Panax ginseng* is noted for broad pharmacological and immune-modulating effects [14,15]. Within ginseng constituents, ginsenoside Rh2 (Rh2) has been investigated for anticancer and immunomodulatory activities across models [16]. Converging evidence suggests that Rh2 can influence inflammation–immunity hubs that also regulate immune-checkpoint programs—including STAT3, NF-κB, and MAPK [6,16,17,18]. However, its immunomodulatory mechanisms remain incompletely understood—particularly under physiologically relevant cancer–immune conditions—because most prior Rh2 studies have relied on cancer cell monocultures that do not recapitulate the complexity of the cancer–immune microenvironment [18].

Therefore, we employed an ex vivo co-culture of murine HCC cells (Hepa1-6) with primary splenocytes to better approximate a suppressive cancer–immune microenvironment. In this physiologically relevant setting, we examined how Rh2 modulates cancer–immune crosstalk, focusing on immune cell activation, cytokine profiles, checkpoint expression, and cancer cell apoptosis.

## 2. Materials and Methods

### 2.1. Cell Culture

Murine hepatocellular carcinoma (Hepa1-6) and hepatocyte (AML12) cell lines (ATCC, Manassas, VA, USA) were maintained in DMEM (Gibco, Waltham, MA, USA) supplemented with 10% fetal bovine serum (FBS; Gibco) and 1% penicillin–streptomycin (Gibco). For AML12 cells, the medium was further supplemented with 1% insulin–transferrin–selenium (Gibco) and 40 ng/mL dexamethasone (Sigma-Aldrich, St. Louis, MO, USA). These media were used only for routine cell maintenance and expansion before the experiments. Cells were incubated at 37 °C in a humidified atmosphere with 5% CO_2_.

### 2.2. Mouse Splenocyte Isolation

Spleens were harvested from 20-week-old male C57BL/6N mice (Central Lab. Animal Inc., Seoul, Republic of Korea). After mechanical dissociation through a 40 μm cell strainer (Corning, Steuben County, NY, USA), erythrocytes were removed using Red Blood Cell Lysing Buffer (Sigma-Aldrich). Splenocytes were washed and resuspended in RPMI-1640 (Gibco) supplemented with 10% FBS and 1% penicillin–streptomycin. Splenocytes were isolated from individual mice and processed separately for all subsequent experiments. All animal procedures were approved by the Institutional Animal Care and Use Committee of Chonnam National University (Approval number: CNU IACUC-YB-2024-137) and conducted in accordance with the ethical guidelines for animal research of the university.

### 2.3. Co-Culture System and Rh2 Treatment

Target cells were seeded into appropriate plates and co-cultured the following day with freshly isolated splenocytes at a 1:80 (target:splenocyte) ratio in a 1:1 mixture of DMEM and RPMI-1640 containing 10% FBS and 1% penicillin–streptomycin. The 1:80 target cell-to-splenocyte ratio was selected based on preliminary optimization and previous splenocyte-based cytotoxicity assays [19]. Ginsenoside Rh2 (≥97% purity; Sigma-Aldrich, USA), which has limited aqueous solubility, was dissolved in DMSO to prepare a 5 mM stock solution and subsequently diluted in culture medium to achieve final concentrations of 0–40 μM. The final DMSO concentrations were 0.2%, 0.4%, and 0.8% for 10, 20, and 40 μM Rh2, respectively. Vehicle controls contained equivalent DMSO concentrations. After establishing the co-culture and applying treatments, cells were incubated for 42 h prior to endpoint analyses. A schematic overview of the co-culture procedure is provided in (Supplementary Figure S1).

### 2.4. Cell Viability and DAPI Staining

For viability assays, AML12 and Hepa1-6 cells were seeded at 5 × 10^3^ cells/well in 96-well plates (Corning) and treated with Rh2 (0–40 μM) under mono- or co-culture conditions. Cell viability was assessed 42 h after treatment using the Ez-Cytox Cell Viability Assay Kit (Daeil Lab Service Co., Ltd., Yongin, Republic of Korea) according to the manufacturer’s protocol. For nuclear morphology analysis, cells were seeded at 4 × 10^4^ cells/well on four-well chamber slides, treated with 10 μM Rh2 or vehicle, fixed with 4% paraformaldehyde, permeabilized with 0.3% Triton X-100, and stained using ProLong™ Gold Antifade Mountant with DAPI (Invitrogen, Bend, OR, USA). Under co-culture conditions, non-adherent splenocytes were gently removed by washing prior to fixation, and nuclear morphology was subsequently evaluated in the remaining adherent Hepa1-6 or AML12 cell population. Fluorescent images were acquired using a fluorescence microscope (OLYMPUS, Tokyo, Japan). Nuclear morphology was evaluated qualitatively using DAPI staining.

### 2.5. Flow Cytometry

For flow cytometry, 2 × 10^5^ target cells/well were seeded in six-well plates and co-cultured with splenocytes as above. After 42 h of treatment, cells were harvested and processed for analysis on a CytoFLEX flow cytometer (Beckman Coulter, Brea, CA, USA), with data analyzed using CytExpert software (v2.3.1.22). Apoptotic cells were detected using the FITC Annexin V Apoptosis Detection Kit I (BD Biosciences, San Jose, CA, USA). For immune profiling, cells were stained with the LIVE/DEAD™ Fixable Aqua Dead Cell Stain Kit, for 405 nm excitation (Invitrogen, USA), then fixed and permeabilized using the Fixation/Permeabilization Buffer Set (Enzo Life Sciences, Farmingdale, NY, USA), followed by antibody staining.

Antibodies used included: CD3 (eFluor 450; Invitrogen), CD4 (FITC; SouthernBiotech, Birmingham, AL, USA), CD8 (PE/Cy5.5; SouthernBiotech), CD25 (PE; R&D Systems, Minneapolis, MN, USA), CD45 (Alexa Fluor 700; Invitrogen), CD107a (PE; Invitrogen), CD154 (eFluor 450; Invitrogen), NK1.1 (FITC; Invitrogen), PD-1 (APC-eFluor 780; Invitrogen), PD-L1 (APC; Lifespan Bioscience, Newark, CA, USA), FOXP3 (APC; R&D Systems), IFN-γ (APC; Invitrogen), IL-6 (eFluor 450; Invitrogen), IL-10 (PerCP-Cy5.5; Invitrogen), IL-12 (APC; BD Biosciences, USA), and TGF-β1 (PE; BioLegend, San Diego, CA, USA). All antibodies were used at manufacturer-recommended dilutions.

### 2.6. Gating Strategy

Cells were first separated into Hepa1-6 and splenocyte populations based on FSC-A and SSC-A characteristics. Doublets were excluded using FSC-A vs. FSC-H, and viable cells were identified by viability dye exclusion. For immune-cell analysis, CD45^+^ leukocytes were gated from the viable splenocyte population. CD4^+^ T cells were identified as CD3^+^CD4^+^ cells, CD8^+^ T cells as CD3^+^CD8^+^ cells, and NK cells as CD3^−^NK1.1^+^ cells. Activation, checkpoint, and cytokine markers were subsequently analyzed within the indicated cell populations. The complete gating strategy is shown in Supplementary Figure S2.

### 2.7. Statistical Analysis

All data are expressed as mean ± standard deviation (SD). *n* represents the number of independent biological replicates. Comparisons between two groups were made using Student’s *t*-test (SPSS Statistics v23.0; IBM, Armonk, NY, USA), with *p* < 0.05 considered statistically significant.

## 3. Results

### 3.1. Effect of Rh2 on Immune-Mediated Cytotoxicity Against Hepa1-6 Cells

**Figure 1:**
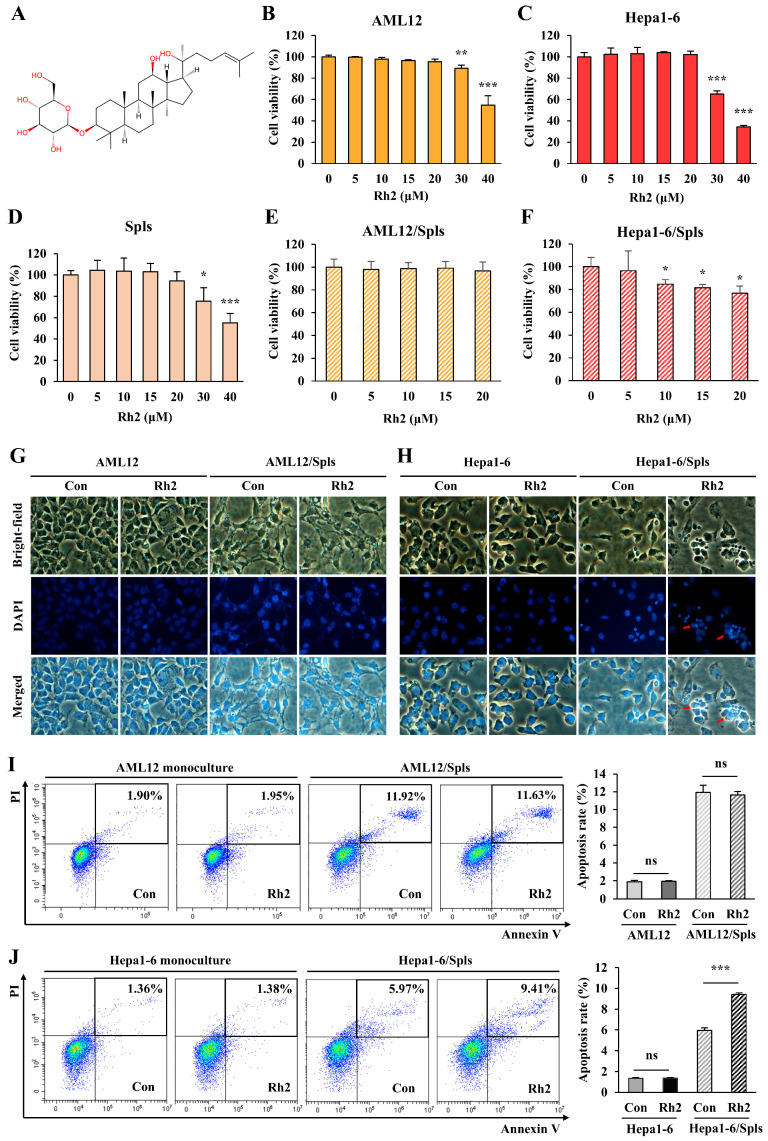
Effect of ginsenoside Rh2 on viability and apoptosis of splenocytes (Spls), AML12 hepatocytes, and Hepa1-6 hepatocellular carcinoma cells under monoculture or co-culture conditions. (**A**) Chemical structure of ginsenoside Rh2. (**B**–**D**) Cell viability of AML12 (**B**), Hepa1-6 (**C**), and splenocytes (**D**) after 42 h treatment with Rh2 (0–40 μM) under monoculture conditions. (**E**,**F**) Viability of AML12 (**E**) and Hepa1-6 (**F**) cells co-cultured with splenocytes and treated with Rh2 (0–20 μM) for 42 h. (**G**,**H**) Bright-field and DAPI-stained images of AML12 (**G**) and Hepa1-6 (**H**) cells treated with or without 10 μM Rh2 under monoculture and co-culture conditions; red arrows indicate apoptotic nuclei. (**I**,**J**) Flow cytometric analysis of Annexin V/PI-stained AML12 (**I**) and Hepa1-6 (**J**) cells under monoculture and co-culture conditions after 42 h treatment with 10 μM Rh2; bar graphs show quantification of apoptotic cells. Data are presented as mean ± SD from three independent biological replicates (*n* = 3). * *p* < 0.05, ** *p* < 0.01, *** *p* < 0.001 vs. control; ns, not significant.

To evaluate the baseline cytotoxicity of Rh2, AML12 hepatocytes, Hepa1-6 hepatocellular carcinoma cells, and splenocytes (Spls) were treated with Rh2 (0–40 μM) for 42 h. Up to 20 μM, cell viability remained stable across all three cell types, indicating that Rh2 does not exert direct cytotoxic effects under monoculture conditions (Figure 1B–D). To assess whether Rh2 modulates immune-mediated cytotoxicity, AML12 and Hepa1-6 cells were co-cultured with Spls and treated with Rh2 (0–20 μM). Rh2 treatment did not significantly affect the bulk viability readout in the AML12/splenocyte co-culture (Figure 1E), whereas a significant decrease in viability was observed in the Hepa1-6/splenocyte co-culture starting at 10 μM Rh2 (Figure 1F). However, since viability assays in co-culture systems do not distinguish between target and effector cell death, further analyses were required to determine whether the reduction reflects selective cytotoxicity against cancer cells.

To visualize cytotoxic effects, nuclear morphology was assessed via DAPI staining. In AML12 cells co-cultured with splenocytes, modest nuclear shrinkage and condensation were observed, consistent with baseline effector activity (Figure 1G), and Rh2 did not induce additional morphological changes. In contrast, Hepa1-6 cells displayed minimal nuclear changes under co-culture, but exhibited marked nuclear condensation and fragmentation following Rh2 treatment (Figure 1H). These findings were corroborated by Annexin V/PI staining, which showed a significant increase in the apoptotic fraction within the Hepa1-6 population under co-culture, while no significant changes were detected in AML12 (Figure 1I,J). Together, these results indicate that Rh2 selectively promotes immune-dependent apoptosis in hepatocellular carcinoma cells under co-culture, with no detectable adverse effect on the AML12/splenocyte condition or the splenocyte compartment under the tested conditions.

### 3.2. Identification of Effector Populations Underlying Rh2-Enhanced Cytotoxicity in Cancer–Immune Co-Culture

Having established that Rh2 selectively enhances immune-mediated apoptosis in hepatocellular carcinoma cells under co-culture conditions, we next sought to identify the immune effector populations responsible for this enhanced cytotoxicity. To do so, we first examined early immune activation markers related to antigen presentation and CD4^+^ T cell priming. We assessed IL-12 expression in total splenocytes and CD154 expression in CD4^+^ T cells by flow cytometry (Supplementary Figure S3). In splenocyte monoculture, IL-12^+^ cells were detected at low frequency and were not significantly altered by Rh2 treatment (Supplementary Figure S3A). Co-culture with AML12 or Hepa1-6 cells led to a modest increase in IL-12^+^ splenocytes, with no significant difference between vehicle- and Rh2-treated groups (Supplementary Figure S3B,C). Similarly, CD154 expression in CD4^+^ T cells increased upon co-culture with AML12 or Hepa1-6 cells, consistent with T cell activation in response to cell–cell interaction; Rh2 did not significantly affect CD154 expression under any condition tested (Supplementary Figure S3D–F).

**Figure 2:**
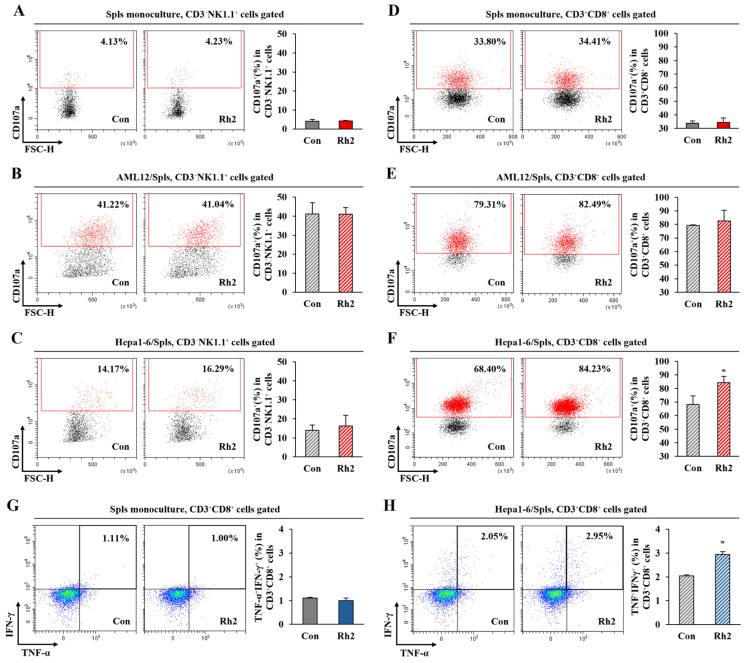
Effect of ginsenoside Rh2 on activation marker expression in NK and CD8^+^ T cells from splenocytes (Spls) under monoculture or co-culture conditions. (**A**–**C**) Representative flow cytometry dot plots with corresponding bar-graph quantification of CD107a^+^ NK cells (CD3^−^NK1.1^+^) from Spls cultured alone (**A**), co-cultured with AML12 hepatocytes (**B**), or co-cultured with Hepa1-6 hepatocellular carcinoma cells (**C**) after 42 h treatment with vehicle (Con) or Rh2 (10 μM). (**D**–**F**) Representative dot plots with bar-graph quantification of CD107a^+^ CD8^+^ T cells (CD3^+^CD8^+^) from Spls cultured alone (**D**), co-cultured with AML12 hepatocytes (**E**), or co-cultured with Hepa1-6 hepatocellular carcinoma cells (**F**) after 42 h treatment with vehicle (Con) or Rh2 (10 μM). (**G**,**H**) Representative dot plots with bar-graph quantification of IFN-γ^+^TNF-α^+^ CD8^+^ T cells from Spls cultured alone (**G**) or co-cultured with Hepa1-6 cells (**H**) after treatment with vehicle (Con) or Rh2 (10 μM) for 42 h. Data are presented as mean ± SD from three independent biological replicates (*n* = 3). * *p* < 0.05 vs. control.

Next, to evaluate the activation of cytotoxic lymphocytes, CD107a expression—a marker of degranulation—was analyzed in NK and CD8^+^ T cells. In splenocyte monoculture, Rh2 treatment did not affect CD107a levels in either NK or CD8^+^ T cell subsets (Figure 2A,D). Under AML12/splenocyte co-culture, both NK and CD8^+^ T cells exhibited increased CD107a expression, which was not further increased by Rh2 (Figure 2B,E). In contrast, in the Hepa1-6/splenocyte co-culture, CD8^+^ T cells displayed notably elevated CD107a levels after Rh2 exposure (Figure 2C). Although NK cells demonstrated a mild elevation in degranulation, this difference did not reach statistical significance (Figure 2F). While both cell types responded under co-culture conditions, CD8^+^ T cells showed more pronounced activation, prompting further investigation of their functional state.

To assess the effector function of CD8^+^ T cells, intracellular cytokine staining was performed. In splenocyte monoculture, Rh2 treatment had no significant effect on the expression of IFN-γ or TNF-α in CD8^+^ T cells (Figure 2G and Supplementary Figure S4A,C). However, under Hepa1-6/splenocyte co-culture, Rh2 significantly increased the proportion of IFN-γ^+^TNF-α^+^ CD8^+^ T cells (Figure 2H), with a corresponding increase in IFN-γ^+^ single-positive cells (Supplementary Figure S4B). TNF-α^+^ single-positive cells remained unchanged (Supplementary Figure S4D). Together, these results indicate that Rh2 promotes cytotoxicity primarily through enhanced activation of CD8^+^ T cells in the cancer–immune co-culture context.

### 3.3. Effect of Rh2 on PD-1/PD-L1 Immune Checkpoint Axis During Cancer–Immune Cell Interaction

**Figure 3:**
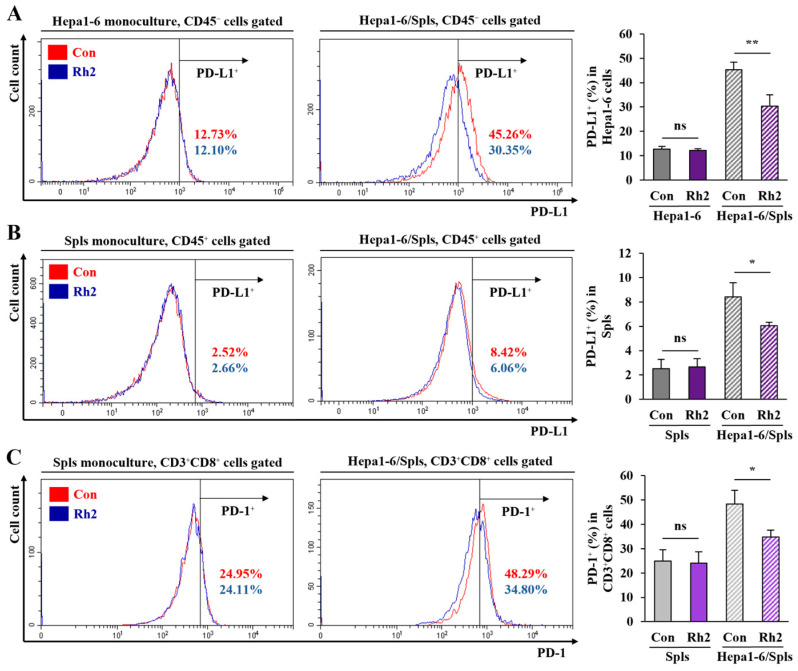
Effect of ginsenoside Rh2 on PD-L1 and PD-1 expression in Hepa1-6 hepatocellular carcinoma cells, splenocytes (Spls), and CD8^+^ T cells. (**A**,**B**) Representative histogram plots with bar-graph quantification of PD-L1^+^ Hepa1-6 cells (**A**) and CD45^+^ Spls (**B**) after 42 h treatment with vehicle (Con) or Rh2 (10 μM) under monoculture or co-culture conditions. (**C**) Representative histograms with quantification of PD-1^+^ CD8^+^ T cells from Spls cultured alone or co-cultured with Hepa1-6 cells under the same treatment conditions. Data are presented as mean ± SD from three independent biological replicates (*n* = 3). * *p* < 0.05, ** *p* < 0.01 vs. control; ns, not significant.

To explore the mechanism underlying the enhanced CD8^+^ T cell-mediated cytotoxicity observed in co-culture, we examined whether Rh2 modulates expression of immune checkpoint molecules. PD-L1 expression in Hepa1-6 cells was low under monoculture conditions and remained unchanged following Rh2 treatment. In co-culture with splenocytes, however, PD-L1 expression was significantly increased and was suppressed by Rh2 treatment (Figure 3A). A similar trend was observed in splenocytes: PD-L1 levels were unaffected in monoculture but elevated upon co-culture with Hepa1-6 cells, with Rh2 reducing this induction (Figure 3B). PD-1 expression in CD8^+^ T cells followed a comparable pattern: it remained low and unaltered in splenocyte monoculture, while co-culture with Hepa1-6 cells led to a marked increase in PD-1^+^ CD8^+^ T cells that was significantly reduced by Rh2 treatment (Figure 3C). These findings suggest that Rh2 may alleviate the immunosuppressive loop mediated by PD-L1/PD-1 signaling in the cancer–immune co-culture context [6,20].

### 3.4. Effect of Rh2 on Immunosuppressive Mechanisms During Cancer–Immune Cell Interaction

**Figure 4:**
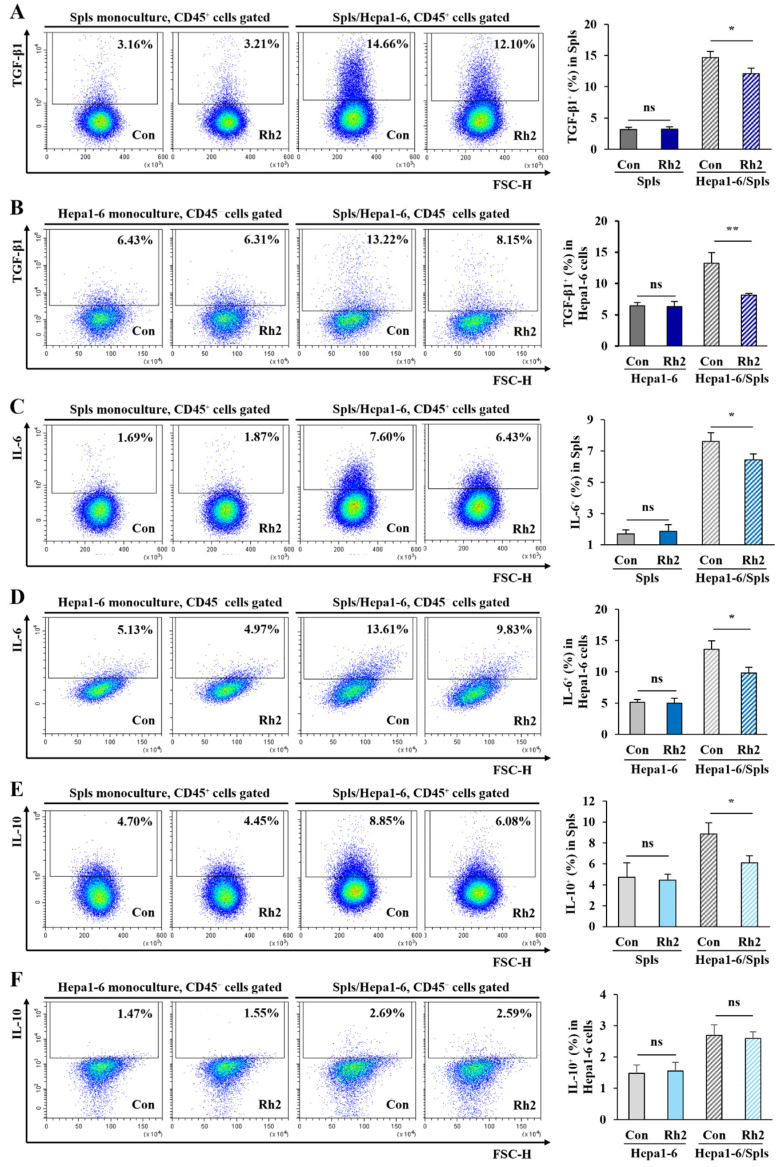
Effect of ginsenoside Rh2 on the expression of immunosuppressive cytokines in splenocytes (Spls) and Hepa1-6 hepatocellular carcinoma cells. (**A**,**B**) Representative flow cytometry dot plots with corresponding bar-graph quantification of TGF-β1^+^ Spls (**A**) and Hepa1-6 cells (**B**) after treatment with vehicle (Con) or Rh2 (10 μM) for 42 h under monoculture or co-culture conditions. (**C**,**D**) Representative dot plots with quantification of IL-6^+^ Spls (**C**) and Hepa1-6 cells (**D**) under the same culture conditions as in (**A**,**B**). (**E**,**F**) Representative dot plots with quantification of IL-10^+^ Spls (**E**) and Hepa1-6 cells (**F**) under the same culture conditions as in (**A**,**B**). Data are presented as mean ± SD from three independent biological replicates (*n* = 3). * *p* < 0.05, ** *p* < 0.01 vs. control; ns, not significant.

**Figure 5:**
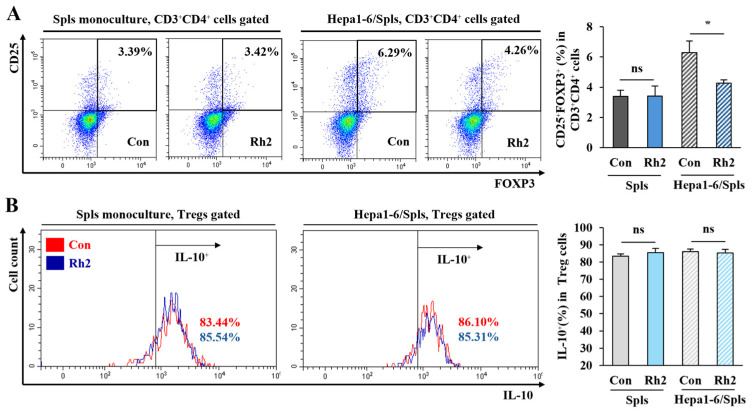
Effect of ginsenoside Rh2 on the regulatory T cell (Treg) population and IL-10 expression in splenocytes (Spls) and Hepa1-6 hepatocellular carcinoma cells. (**A**) Representative flow cytometry dot plots with bar-graph quantification of CD25^+^FOXP3^+^ Tregs among CD4^+^ T cells after 42 h treatment with vehicle (Con) or Rh2 (10 μM) in Spls cultured alone or co-cultured with Hepa1-6 cells. (**B**) Representative histogram plots with quantification of IL-10^+^ cells within the Treg population under the same culture and treatment conditions. Data are presented as mean ± SD from three independent biological replicates (*n* = 3). * *p* < 0.05 vs. control; ns, not significant.

To explore potential mechanisms underlying Rh2-induced CD8^+^ T cell activation and the reduction in PD-L1/PD-1 expression, we examined key immunosuppressive factors. Specifically, we assessed the expression of regulatory cytokines (TGF-β1, IL-6, IL-10) and the frequency of Tregs under cancer–immune co-culture conditions. Under Hepa1-6/splenocyte co-culture, TGF-β1 expression was significantly elevated in both splenocytes and Hepa1-6 cells, compared to their respective monocultures (Figure 4A,B). Rh2 treatment notably reduced TGF-β1 expression under co-culture conditions in both cell types. Similarly, IL-6 levels were increased by co-culture and were significantly suppressed by Rh2 in both Hepa1-6 cells and splenocytes (Figure 4C,D). IL-10 expression in Hepa1-6 cells remained low and unchanged across conditions (Figure 4F), while splenocytes exhibited a significant reduction in IL-10^+^ cells upon Rh2 treatment during co-culture (Figure 4E). We next examined the frequency of Tregs (CD25^+^FOXP3^+^ among CD4^+^ T cells) and their IL-10 production. Hepa1-6/splenocyte co-culture markedly increased the proportion of Tregs, which was significantly reduced by Rh2 treatment (Figure 5A). However, the proportion of IL-10^+^ cells within the Treg population remained unchanged regardless of treatment (Figure 5B). Together, these results indicate that Rh2 attenuates multiple immunosuppressive components—namely TGF-β1, IL-6, IL-10 and Treg expansion—within the cancer–immune interface, potentially contributing to the restoration of anti-cancer–immune responses [5].

## 4. Discussion

Rh2 is a bioactive metabolite from *Panax ginseng* with documented anticancer activities—including apoptosis induction, cell-cycle arrest, and modulation of oncogenic signaling—yet most prior work has emphasized cancer-intrinsic mechanisms, leaving its effects at the cancer–immune interface less well defined [16,18]. To address this gap, we employed an ex vivo co-culture system of murine splenocytes and Hepa1-6 hepatocellular carcinoma cells, with AML12 hepatocytes analyzed in parallel for comparison, to evaluate whether Rh2 modulates immune responses in a cancer-associated setting.

Rh2 did not affect cell viability under monoculture conditions up to 20 μM but selectively reduced viability in Hepa1-6 cells under co-culture conditions, while having no effect on AML12 cells, indicating an immune-dependent, context-specific rather than indiscriminate effect. This differential response between AML12 and Hepa1-6 cells reflects cancer-associated immunosuppression that can be alleviated by Rh2 [7,18]. When splenocytes were co-cultured with either AML12 or Hepa1-6, baseline cytotoxicity was stronger toward AML12—consistent with cell-line-intrinsic properties and, potentially, immune-evasion mechanisms in cancer cells influencing baseline reactivity [8,21,22,23]. Under these conditions, Rh2 selectively enhanced immune-mediated killing in the Hepa1-6/splenocyte setting, shifting the balance toward effective cytotoxicity in the HCC context—a pattern aligned with mechanisms commonly described for immunosuppressive cancer–immune microenvironments [24,25]. Accordingly, subsequent analyses focus on the Hepa1-6 context to delineate how Rh2 modulates cancer–immune crosstalk.

We assessed immune effector function, focusing on early activation markers and indicators of cytotoxic activity, to elucidate mechanisms of Rh2-mediated immune potentiation. Rh2 did not affect early activation markers such as IL-12 in bulk splenocytes or CD154 in CD4^+^ T cells, implying minimal impact on antigen-presenting cell (APC)-mediated T cell priming [26,27]. By contrast, in CD8^+^ T cells, Rh2 significantly increased the frequency of CD107a^+^ cells, indicating enhanced degranulation.

In parallel, intracellular cytokine analysis showed a clear increase in IFN-γ^+^ CD8^+^ T cells and an expansion of IFN-γ^+^TNF-α^+^ double-positive subsets. Together, increased CD107a and IFN-γ indicate strengthened cytotoxic effector responses in CD8^+^ T cells following Rh2 treatment [28,29,30].

Given the enhanced CD8^+^ T cell activity observed under co-culture conditions, we evaluated whether Rh2 modulates the PD-L1/PD-1 axis, a key inhibitory pathway in the cancer–immune microenvironment [31]. PD-L1 expression was elevated in both Hepa1-6 cells and splenocytes upon co-culture, consistent with a response to cancer–immune interactions [8,32], and Rh2 downregulated this induction in both compartments during co-culture. On CD8^+^ T cells, PD-1 was likewise induced by co-culture with Hepa1-6 and was significantly reduced by Rh2; this pattern is compatible with a bidirectional suppressive loop between PD-L1 and PD-1 at the cancer–immune interface [8,20]. These findings support the notion that Rh2 mitigates immune-checkpoint signaling specifically under suppressive cancer–immune conditions, thereby enhancing CD8^+^ T cell responsiveness.

To further dissect the immunosuppressive landscape targeted by Rh2, we examined its effects on cytokines and regulatory cell populations that shape the cancer–immune microenvironment. Under co-culture, TGF-β1, IL-6, and IL-10 were elevated, consistent with a suppressive milieu known to impair effector T cell function and sustain Treg populations [7,33]. Rh2 treatment suppressed TGF-β1 and IL-6 in both Hepa1-6 and splenocytes compartments. By contrast, IL-10—also reported from hepatocytes [34,35]—was selectively reduced in splenocytes, while hepatocyte-derived IL-10 remained unchanged. This cytokine shift was accompanied by a decrease in CD4^+^CD25^+^FOXP3^+^ Tregs under co-culture. Notably, although Rh2 reduced the overall frequencies of IL-10^+^ splenocytes and Tregs, the fraction of IL-10^+^ cells within the Treg gate was unchanged, suggesting that the decline in total IL-10^+^ cells primarily reflects reduced Treg abundance rather than altered IL-10 production per Treg. Given that TGF-β1 and IL-10 are key drivers of Treg differentiation and maintenance [36,37], these findings suggest that Rh2 may limit Treg expansion, at least in part, through suppression of upstream immunosuppressive cytokines.

Several limitations of the present study should be considered. First, secreted cytokine concentrations in culture supernatants were not measured. Future studies using ELISA or multiplex cytokine analyses will be required to validate whether the observed intracellular changes are reflected at the extracellular level. In addition, whole splenocytes were used rather than purified immune-cell subsets, limiting the ability to define the specific contribution of individual immune-cell populations. Furthermore, because the present findings were obtained using an ex vivo co-culture model, additional in vivo studies will be required to confirm the immunomodulatory effects of Rh2 within the complex tumor microenvironment. Despite these limitations, the present findings provide evidence that Rh2 modulates cancer–immune interactions and enhances antitumor immune responses in an HCC–splenocyte co-culture system.

## 5. Conclusions

In this co-culture model, Rh2 appears to modulate multiple tiers of the immunosuppressive network within the cancer–immune microenvironment. It is associated with enhanced CD8^+^ T cell effector function, attenuation of the PD-L1/PD-1 axis, reductions in TGF-β1 and IL-6, and a contraction of regulatory T cells. These findings suggest that Rh2 may enhance anticancer immunity through multi-target immunomodulation.

## Data Availability

The original contributions presented in this study are included in the article/Supplementary Materials. Further inquiries can be directed to the corresponding author.

## Supplementary Materials

The following supporting information can be downloaded at: https://www.mdpi.com/article/10.3390/nu18142224/s1, Figure S1: Schematic illustration of the co-culture workflow. Target cells (AML12 or Hepa1-6) were seeded and pre-incubated overnight. Primary mouse splenocytes were then added, followed by treatment with ginsenoside Rh2 or vehicle. After 42 h, cellular responses were assessed by WST assay, DAPI staining, and flow cytometry. Figure S2: Gating strategy for flow cytometric analysis of co-cultured cells. Flow cytometry data from co-culture experiments were analyzed using a standardized gating strategy to define immune cell subsets. Cells were first gated using forward and side scatter (FSC-A vs. SSC-A) to differentiate populations by size and granularity. Singlets were identified using FSC-A vs. FSC-H to exclude doublets and aggregates. Viable cells were selected by viability-dye staining, excluding dead cells. To distinguish immune cells from target cells in co-culture, lineage-specific surface markers (CD45, CD3, CD8, NK1.1) were used. Subsequent analyses on these immune subsets evaluated functional markers, including activation molecules, immune checkpoints, intracellular cytokines, and sub-population characteristics. Figure S3: Effect of ginsenoside Rh2 on IL-12 and CD154 expression in splenocytes (Spls) and CD4^+^ T cells under monoculture or co-culture conditions. (A–C) Representative flow cytometry dot plots with corresponding bar-graph quantification of IL-12^+^ Spls following 42 h treatment with vehicle (Con) or Rh2 (10 µM). Spls were cultured alone (A), co-cultured with AML12 hepatocytes (B), or co-cultured with Hepa1-6 hepatocellular carcinoma cells (C). (D–F) Representative dot plots with corresponding quantification of CD154^+^ CD4^+^ T cells under the same culture conditions as in (A–C). Data are presented as mean ± SD (*n* = 3). * *p* < 0.05, ** *p* < 0.01, ** *p* < 0.001 vs. control; ns, not significant. Figure S4: Effect of ginsenoside Rh2 on IFN-γ and TNF-α expression in CD8^+^ T cells from splenocytes (Spls) under monoculture or co-culture conditions. (A–B) Bar-graph quantification of IFN-γ^+^ CD8^+^ T cells from Spls cultured alone (A) or co-cultured with Hepa1-6 cells (B) following treatment with vehicle (Con) or Rh2 (10 μM) for 42 h. (C–D) Bar-graph quantification of TNF-α^+^ CD8^+^ T cells under the same respective conditions (C,D). Data are presented as mean ± SD (*n* = 3). *p* < 0.05 vs. control; ns, not significant.
