# Supplementary material for: Ginsenoside Rh2 Enhances CD8+ T Cell-Mediated Anticancer Immunity in Hepatocellular Carcinoma
Source: Nutrients. 2026 Jul 8;18(14):2224. doi: 10.3390/nu18142224 (PMC13415868; doi:10.3390/nu18142224)
Supplement: Supplementary file 1 [file nutrients-18-02224-s001.zip › nutrients-4372016-supplementary.pdf]

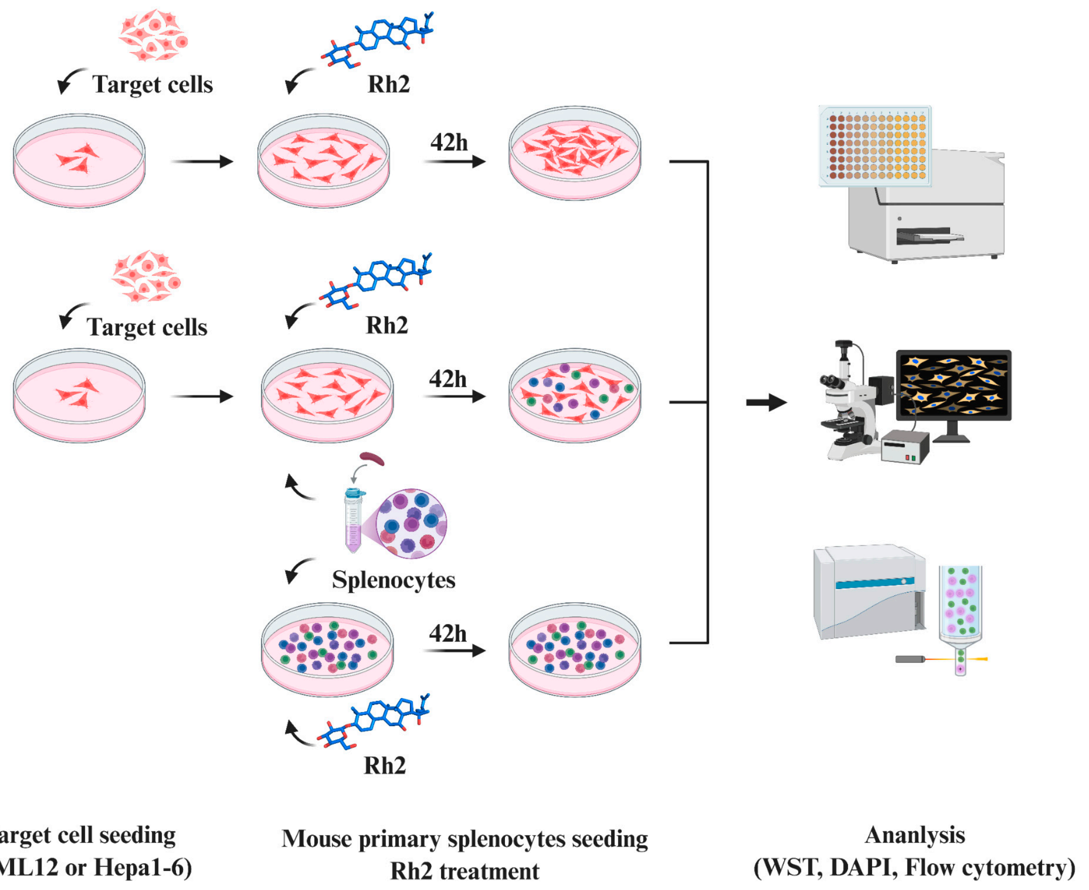

Supplementary Figure S1. Schematic illustration of the co-culture workflow. Target cells (AML12 or Hepa1-6) were seeded and pre-incubated overnight. Primary mouse splenocytes were then added, followed by treatment with ginsenoside Rh2 or vehicle. After 42 h, cellular responses were assessed by WST assay, DAPI staining, and flow cytometry.

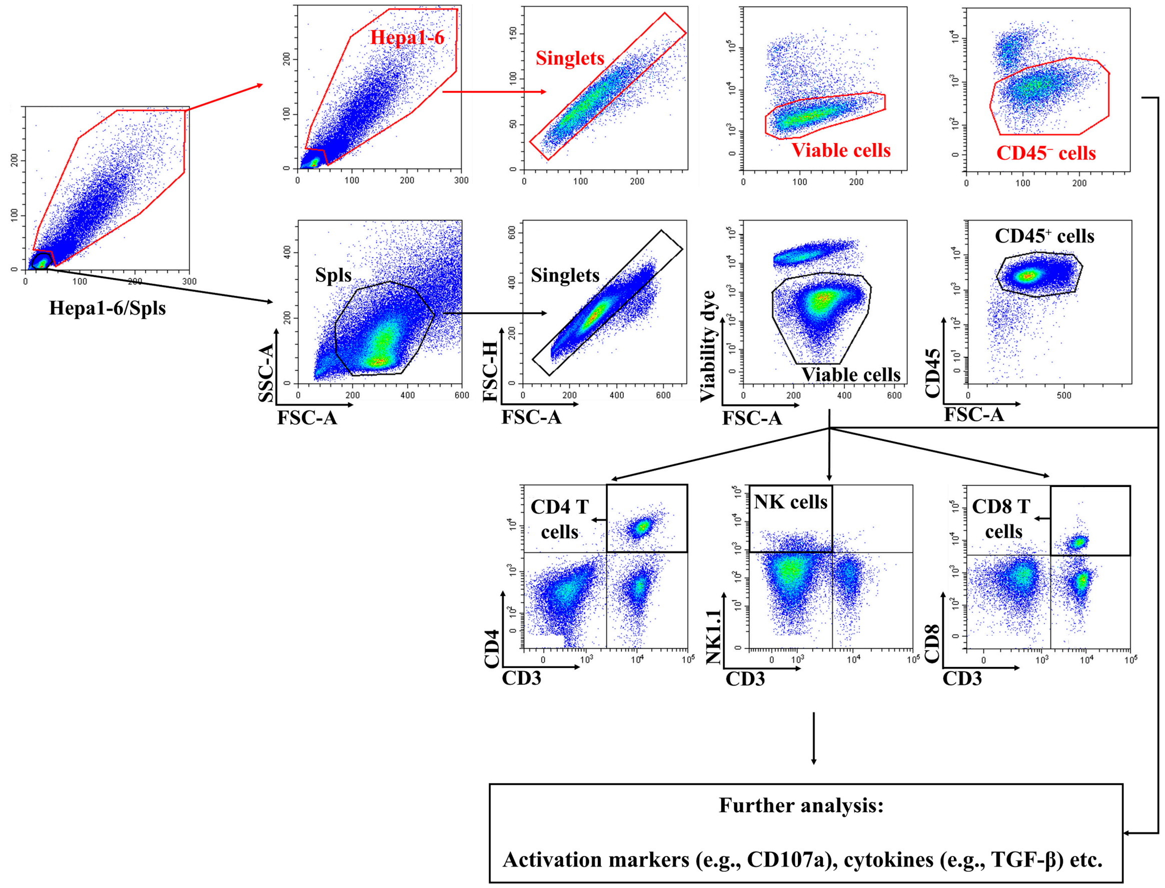

Supplementary Figure S2. Gating strategy for flow cytometric analysis of co-cultured cells. Flow cytometry data from co-culture experiments were analyzed using a standardized gating strategy to define immune cell subsets. Cells were first gated using forward and side scatter (FSC-A vs. SSC-A) to differentiate populations by size and granularity. Singlets were identified using FSC-A vs. FSC-H to exclude doublets and aggregates. Viable cells were selected by viability-dye staining, excluding dead cells. To distinguish immune cells from target cells in co-culture, lineage-specific surface markers (CD45, CD3, CD8, NK1.1) were used. Subsequent analyses on these immune subsets evaluated functional markers, including activation molecules, immune checkpoints, intracellular cytokines, and sub-population characteristics.

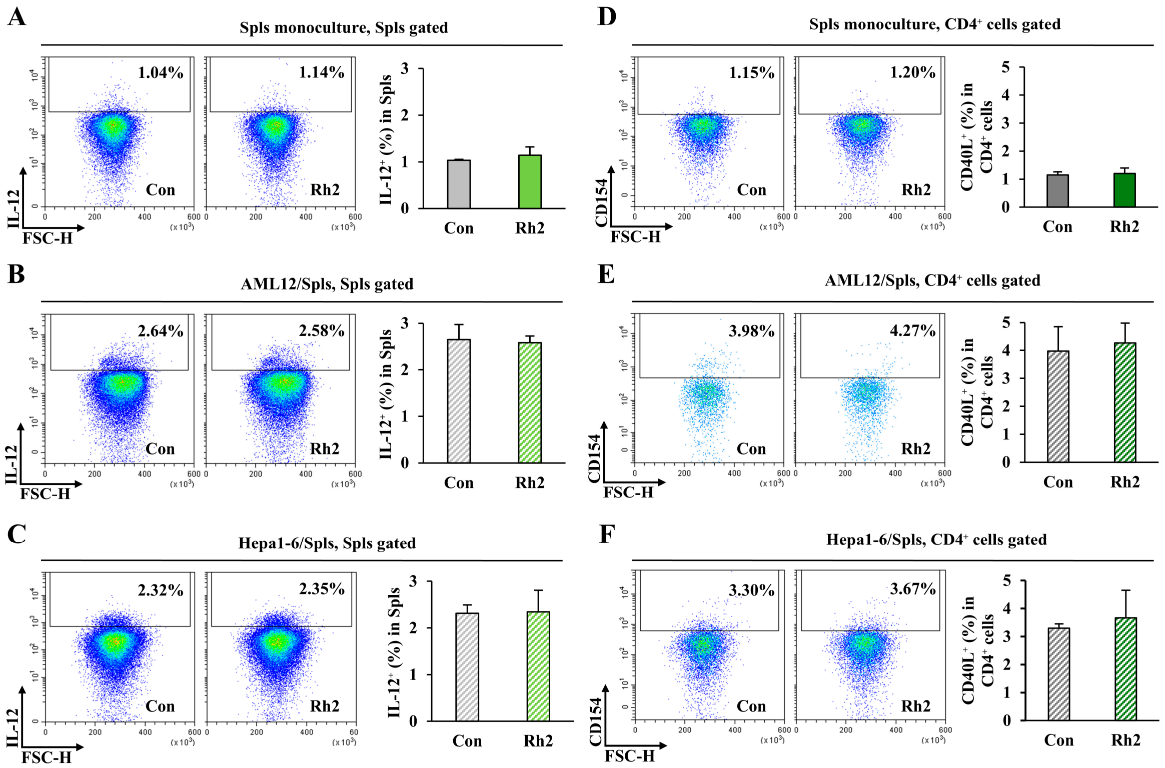

Supplementary Figure S3. Effect of ginsenoside Rh2 on IL-12 and CD154 expression in splenocytes (Spls) and CD4$^+$ T cells under monoculture or co-culture conditions. (A–C) Representative flow cytometry dot plots with corresponding bar-graph quantification of IL-12$^+$ Spls following 42 h treatment with vehicle (Con) or Rh2 (10 μM). Spls were cultured alone (A), co-cultured with AML12 hepatocytes (B), or co-cultured with Hepa1-6 hepatocellular carcinoma cells (C). (D–F) Representative dot plots with corresponding quantification of CD154$^+$ CD4$^+$ T cells under the same culture conditions as in (A–C). Data are presented as mean ± SD (n = 3). *$p < 0.05$, **$p < 0.01$, **$p < 0.001$ vs. control; ns, not significant.

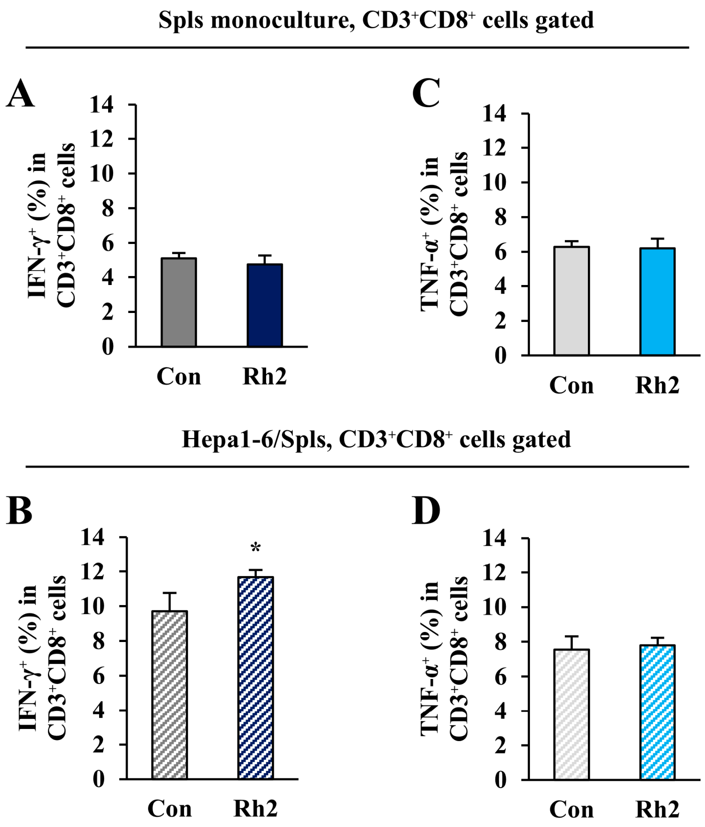

Supplementary Figure S4. Effect of ginsenoside Rh2 on IFN-γ and TNF-α expression in CD8$^+$ T cells from splenocytes (Spls) under monoculture or co-culture conditions. (A–B) Bar-graph quantification of IFN-γ$^+$ CD8$^+$ T cells from Spls cultured alone (A) or co-cultured with Hepa1-6 cells (B) following treatment with vehicle (Con) or Rh2 (10 µM) for 42 h. (C–D) Bar-graph quantification of TNF-α$^+$ CD8$^+$ T cells under the same respective conditions (C, D). Data are presented as mean ± SD (n = 3). $p < 0.05$ vs. control; ns, not significant.
